# Bisphenol A Is More Potent than Phthalate Metabolites in Reducing Pancreatic *β*-Cell Function

**DOI:** 10.1155/2017/4614379

**Published:** 2017-02-13

**Authors:** Nina Mickelson Weldingh, Lena Jørgensen-Kaur, Rune Becher, Jørn A. Holme, Johanna Bodin, Unni C. Nygaard, Anette Kocbach Bølling

**Affiliations:** Domain of Infection Control and Environmental Health, Norwegian Institute of Public Health, P.O. Box 4404, Nydalen, 0403 Oslo, Norway

## Abstract

Bisphenol A (BPA) and phthalates are common environmental contaminants that have been proposed to influence incidence and development of types 1 and 2 diabetes. Thus, effects of BPA and three phthalate metabolites (monoisobutyl phthalate (MiBP), mono-n-butyl phthalate (MnBP), and mono-(2-ethylhexyl) phthalate (MEHP)) were studied in the pancreatic *β*-cell line INS-1E, after 2–72 h of exposure to 5–500 *μ*M. Three endpoints relevant to accelerated development of types 1 or 2 diabetes were investigated: *β*-cell viability, glucose-induced insulin secretion, and *β*-cell susceptibility to cytokine-induced cell death. BPA and the phthalate metabolites reduced cellular viability after 72 h of exposure, with BPA as the most potent chemical. Moreover, BPA, MEHP, and MnBP increased insulin secretion after 2 h of simultaneous exposure to chemicals and glucose, with potency BPA > MEHP > MnBP. Longer chemical exposures (24–72 h) showed no consistent effects on glucose-induced insulin secretion, and none of the environmental chemicals affected susceptibility to cytokine-induced cell death. Overall, BPA was more potent than the investigated phthalate metabolites in affecting insulin secretion and viability in the INS-1E pancreatic *β*-cells. In contrast to recent literature, concentrations with relevance to human exposures (1–500 nM) did not affect the investigated endpoints, suggesting that this experimental model displayed relatively low sensitivity to environmental chemical exposure.

## 1. Introduction

The use of plastic products is widespread, resulting in daily exposure to a range of chemicals that may leak from plastics [1, 2]. These chemicals can be ingested via contaminated food and beverage, absorbed through skin contact, or inhaled [1, 2]. Two major classes of chemicals leaching from plastics are phthalates, used as plasticizers in polyvinylchloride products, and bisphenol A (BPA), used in polycarbonate plastic and epoxy resins. These chemicals are produced in several million tons per year [3, 4] and have been detected in blood and urine samples from the majority of the individuals in investigated populations [3]. In recent years, there has been a growing concern about the adverse health effects of these environmental chemicals, based on knowledge gained from both animal and epidemiological studies [1, 2, 5]. Therefore, regulations have been introduced in the EU and other countries, both with respect to product content and production of phthalates and BPA. However, these chemicals still occur in many consumer products that we are in daily contact with causing ubiquitous exposure [4].

Diabetes is a common disease worldwide, and the substantial economic and social costs associated with the increasing incidence of diabetes represent a major public health issue [6]. Lifestyle changes associated with industrialization and rapid economic development have been suggested to contribute to the increased diabetes incidences seen the last thirty years. Moreover, the substantial increase in human exposure to synthetic chemicals, including endocrine disruptors [7], coincides with the increased diabetes incidence [8]. Accordingly, various environmental chemicals have also been associated with outcomes with relevance to diabetes development [9–12].

Type 1 diabetes (T1D) is an autoimmune disease that usually first occurs in the early childhood or in the young adult years. It is characterized by an extensive loss of the insulin producing pancreatic *β*-cells, causing insulin deficiency, and thus elevated blood glucose levels [13]. Cytokine-induced cell death of pancreatic *β*-cells seems to be involved in this process [14]. In contrast to T1D, type 2 diabetes (T2D) usually develops in the adult age and among the elderly and is considered to account for more than 90% of all cases of diabetes [6]. The main features of T2D are a deficiency in insulin secretion or action, resulting in abnormally high blood and plasma glucose level [15]. Although a relative or absolute deficiency in insulin secretion characterizes T2D, hypersecretion of insulin may occur as an early step in the development of this disease [16].

Epidemiological and experimental studies have linked exposure to both BPA and phthalates to several metabolic effects, including diabetes [12, 17–23]. BPA has been associated with T2D and impaired pancreatic *β*-cell function in experimental studies and increased glucose-induced insulin secretion from pancreatic islets, that is, hypersecretion of insulin [20, 24–26]. There is limited knowledge regarding a possible impact of BPA on T1D, although associations between some environmental chemicals and T1D have been reported [10, 27]. Recently, we showed that both long term and prenatal exposure to BPA accelerated the spontaneous development of T1D in the nonobese diabetic (NOD) mouse model [23, 28], providing further support for a possible association between BPA exposure and T1D development. In contrast, phthalates did not accelerate the T1D development when tested in the same model system [29].

Phthalates have generally received less attention than BPA in relation to both T1D and T2D. However, an increasing number of epidemiological studies report associations between urinary levels of phthalate metabolites and T2D, as well as outcomes associated with T2D such as increased waist circumference, poor insulin secretion, and insulin resistance [12, 17, 30–32]. In contrast, very few experimental studies have investigated phthalate-induced effects in model systems with relevance to diabetes. Gestational exposure to diethylhexyl phthalate (DEHP) in rats has been associated with *β*-cell dysfunction in the offspring, possibly through downregulation of genes by epigenetic mechanisms [22, 33]. Moreover, direct exposure of pancreatic *β*-cells to DEHP caused apoptosis and ER stress as well as altered insulin secretion [34, 35] and low doses of monoethyl phthalate (MEP) triggered proliferation and increased insulin release from human pancreatic beta cells [36]. Although these studies provide some support for the associations reported in the epidemiological studies, there is still limited knowledge concerning effects of phthalates on *β*-cell function. Thus, further experimental studies are necessary to substantiate the associations reported in the epidemiological studies.

Since pancreatic *β*-cells have a central role in the development of diabetes [15], the aim of this study was to compare the potency of the environmental contaminants BPA and phthalates in terms of their effect on the functionality of these cells* in vitro*. The INS-1E rat pancreatic *β*-cell line has previously been used in mechanistic studies of insulin secretion [37] and cytokine-induced cell death [38] but not to study the impact of environmental chemicals on *β*-cell function. Three phthalate metabolites were included, mono-n-butyl phthalate (MnBP), monoisobutyl phthalate (MiBP), and mono-ethyl-hexyl phthalate (MEHP), which are metabolites of di-n-butyl phthalate (DnBP), di-isobutyl phthalate (DiBP), and DEHP, respectively. All of them are found to be major contributors to phthalate exposure through ingestion [3, 39]. The effects of 5–500 *μ*M of these chemicals alone or in combination were investigated after 2 to 72 h of incubation to compare their relative potency. Three endpoints with relevance to accelerated development of T1D or T2D were examined: glucose-induced insulin secretion, *β*-cell viability, and susceptibility to cytokine-induced cell death. Environmentally relevant concentrations (1–500 nM) of the chemicals were also tested in a selection of the examined endpoints.

## 2. Materials and Methods

### 2.1. Chemicals and Reagents

BPA was obtained from TCI Europe (Zwijndrecht, Belgium), while MiBP, MnBP, and MEHP were from Orchid Cellmark (New Westminster, BC, Canada). RPMI 1640 medium with 2 mM glutamine was purchased from Cambrex Bio Science (Verviers, Belgium), fetal bovine serum (FBS) from Euroclone (Italy), and penicillin/streptomycin from BioWhittaker™ (MD, USA). Sodium pyruvate, HEPES, 2-mercaptoethanol, dimethyl sulfoxide (DMSO), 3-isobutyl-1-methylxanthine (IBMX), Forskolin, thiazolyl blue tetrazolium blue, Hoechst 33342, and propidium iodide were purchased from Sigma-Aldrich (MO, USA). The high range rat insulin enzyme-linked immunosorbent assay (ELISA) kit was obtained from Mercodia (Uppsala, Sweden), while rat recombinant IL-1*β*, IFN*γ*, and TNF*α* were purchased from PromoKine (Heidelberg, Germany).

### 2.2. Cell Culturing Conditions

The rat pancreatic *β*-cell line INS-1E, kindly provided by Professor C. B. Wollheim (University of Geneva, Switzerland), was cultured in a humidified atmosphere at 37°C and 5% CO_2_ in RPMI 1640 medium supplemented with 5% heat-inactivated fetal calf serum, 1 mM sodium pyruvate, 50 *μ*M 2-mercaptoethanol, 2 mM glutamine, 10 mM HEPES, 100 U/mL penicillin, and 100 *μ*g/mL streptomycin, as previously described [37].

For environmental chemical exposure, the cells were seeded in 24-well Falcon plates, in 1 mL cell culture medium. To ensure a linear growth phase during the full exposure period, 300.000, 250.000, 200.000, and 120.000–140.000 cells/well were used for the 2, 24, 48, and 72 h exposure durations, respectively. The cells were incubated for 24 h to allow proper attachment and supplied with 1 mL fresh cell culture medium 2 h prior to exposure.

**Figure 1:**
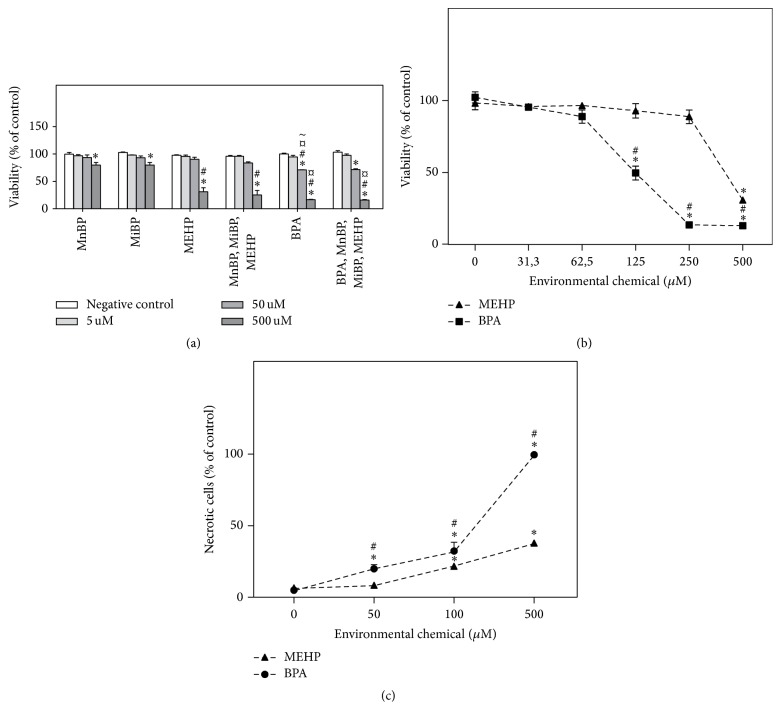
Environmental chemicals affected cell viability and toxicity. INS-1E cells were incubated with the indicated concentrations of environmental chemicals for 72 h in (a) and (b) and 24 h in (c). In the combinatory exposures the indicated doses reflect the concentrations of each chemical rather than the total (additive) chemical concentration. (a and b) The cell viability was measured by the MTT assay and normalized to control, that is, divided by the mean of all the controls in that experiment. (c) The fractions of viable, necrotic, and apoptotic cells were determined by Hoechst/PI staining and fluorescence microscopy, and the percentage corresponding to the sum of necrotic and apoptotic cells is displayed in the figure. The symbols in (a) indicate significant decreases compared to *∗* negative controls or to the corresponding concentrations of # MnBP and MiBP, *¤*  MEHP, and ~ combinatory exposure to MnBP, MiBP, and MEHP (two-way ANOVA with Bonferroni posttest, *N* = 3). In (b and c) *∗* indicates significant difference compared to controls while # indicates significant difference from MEHP at the same concentration (two-way ANOVA with Bonferroni posttest, *N* = 3).

**Figure 2:**
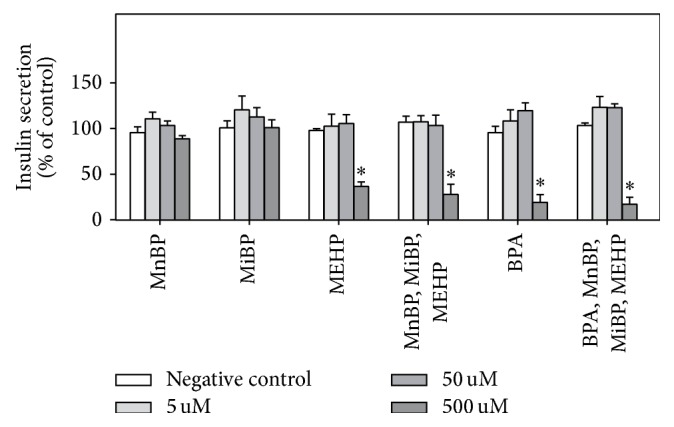
Environmental chemicals reduced the insulin secretion. INS-1E cells were incubated with the indicated concentrations of environmental chemicals for 72 h, incubated with glucose-free KRBH buffer for 1 h followed by 30 min stimulation with 6.7 mM glucose in KRBH buffer. In the combinatory exposures the indicated doses reflect the concentrations of each chemical rather than the total (additive) chemical concentration. The graph shows normalized data, that is, divided by the mean value of the negative controls in that experiment, and *∗* indicates significant decrease compared to control (2-way ANOVA, with Bonferroni posttest, *N* = 4).

**Figure 3:**
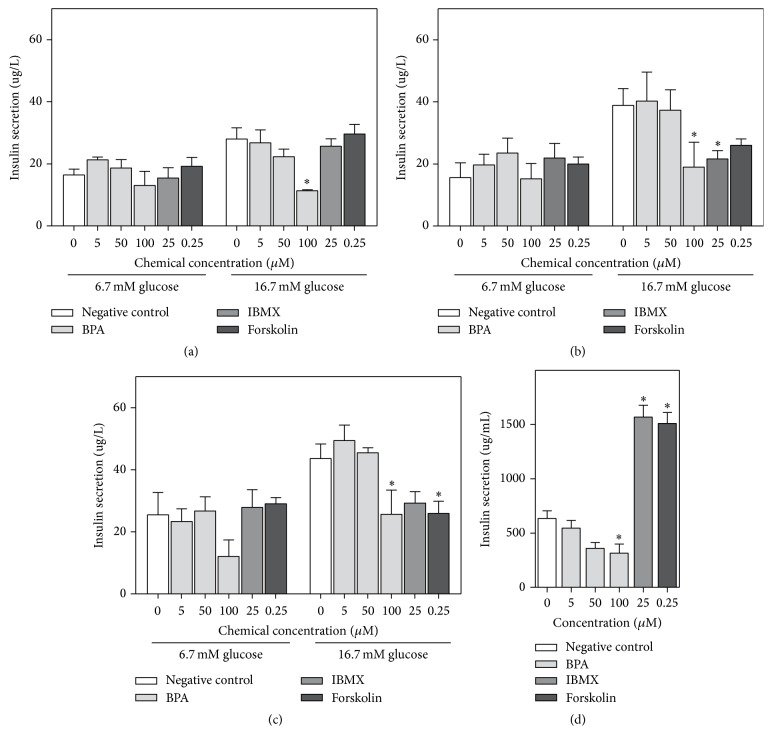
Effect of incubation time and glucose concentration on insulin secretion. INS-1E cells were incubated with the indicated concentrations of BPA or the positive controls IBMX (25 *μ*M) and Forskolin (0.25 *μ*M) for (a) 2 h, (b) 24 h, or (c) 48 h before 1 h incubation with glucose-free KRBH buffer followed by 30 min incubation with 6.7 or 16.7 mM glucose in KRBH buffer. In addition, the insulin levels in the cell culture medium, containing 11.1 mM glucose, were measured after 48 h of stimulus incubation in (d) to test whether the positive control and BPA exposures exhausted the cellular insulin secretion. For these insulin analyses, the medium was harvested prior to the glucose stimulation in KRBH buffer. For all figures, *∗* indicates significant difference from negative control (2-way ANOVA, repeated measures, with Bonferroni posttest, *N* = 3 (in a–c); 1-way ANOVA with Dunnett's posttest, *N* = 4 (in d)).

**Figure 4:**
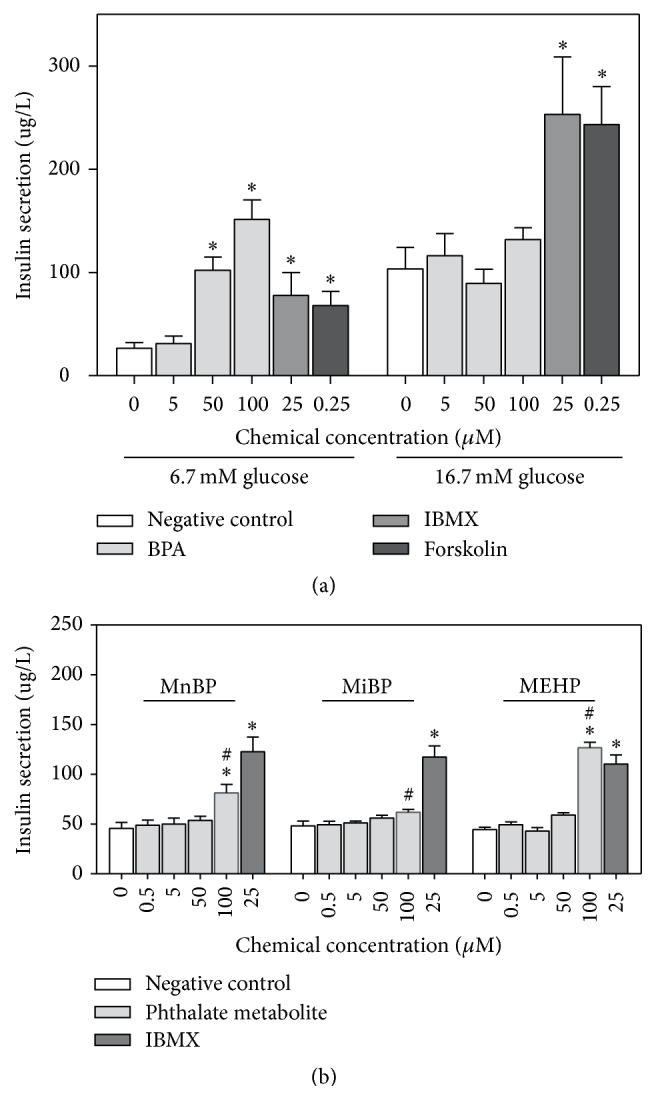
Increased insulin secretion after concomitant exposure to glucose and BPA, phthalate metabolites, or positive controls. INS-1E cells were incubated for 1 h with glucose-free KRBH buffer, followed by 2 h incubation with (a) BPA or the positive controls IBMX (25 *μ*M) and Forskolin (0.25 *μ*M) in the presence of 6.7 or 16.7 mM glucose in KRBH buffer. This concomitant exposure scenario was also used in (b) for the indicated concentrations of phthalate metabolites and the positive control IBMX (25 *μ*M), but only for 6.7 mM glucose. For all figures, *∗* indicates significant increase compared to control, while # indicates significant difference from the other phthalate metabolites at the corresponding concentration (2-way ANOVA, repeated measures, with Bonferroni posttest, *N* = 3-4).

**Figure 5:**
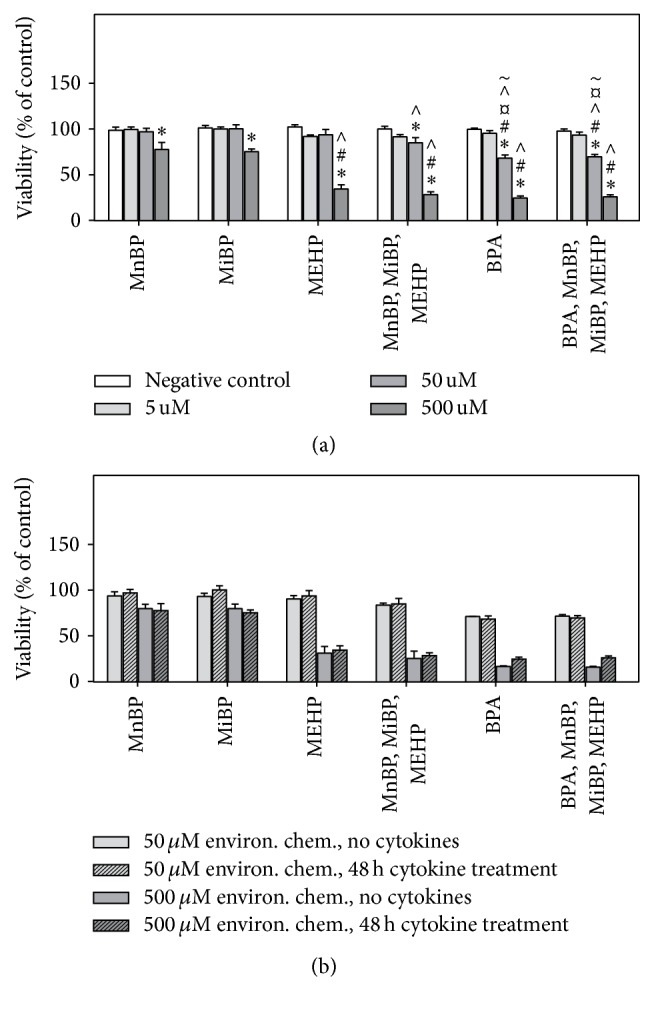
No effect of environmental chemicals on cytokine-induced cell death. (a) INS-1E cells were incubated with the indicated concentrations of environmental chemicals for 72 h with exposure to the proinflammatory cytokines IL-1*β* (5 ng/mL), TNF*α* (25 ng/mL), and INF*γ* (25 ng/mL) the last 48 h of exposure. In the combinatory exposures the indicated doses reflect the concentrations of each chemical rather than the total (additive) chemical concentration. The cell viability was measured by the MTT assay and normalized to control, for example, divided by the mean of all the controls in that experiment. The symbols indicate significant decreases compared to *∗* controls or to the same concentrations of # MnBP, ∧ MiBP, *¤*  MEHP, and ~ combinatory exposure to MnBP, MiBP, and MEHP (two-way ANOVA with Bonferroni posttest, *N* = 3). (b) Comparison of the effects of environmental chemicals in absence and presence of the proinflammatory cytokines. The figure shows data from Figures 1(a) and 5(a) relative to their respective controls. There were no significant differences between the data obtained in absence or presence of cytokines (two-way ANOVA with Bonferroni posttest, *N* = 3).

**Figure 6:**
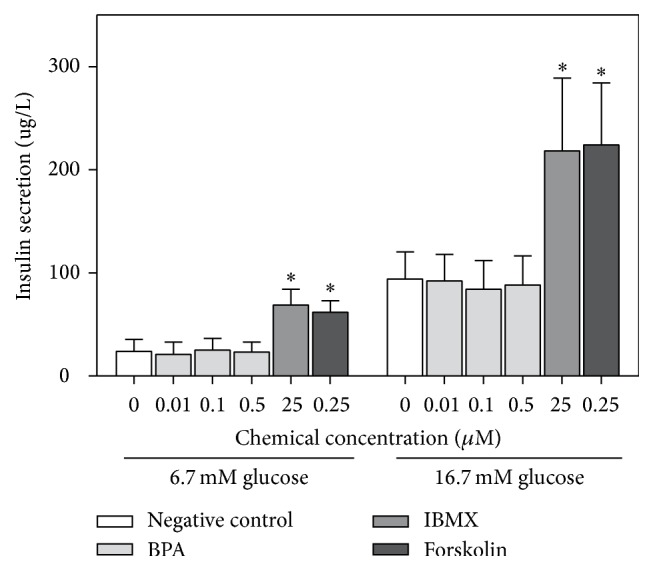
No effect of environmentally relevant BPA concentrations on insulin secretion after concomitant exposure with glucose. INS-1E cells were incubated for 1 h with glucose-free KRBH buffer, followed by 2 h incubation with BPA or the positive controls IBMX (25 *μ*M) and Forskolin (0.25 *μ*M) in the presence of 6.7 or 16.7 mM glucose in KRBH buffer. *∗* indicates significant increase compared to control (2-way ANOVA, repeated measures, with Bonferroni posttest, *N* = 3-4).

The culturing of the cells was performed in medium containing phenol red. For environmental chemical exposure, RPMI medium with phenol red was applied in some series of some experiments (Figures 1, 2, and 5; Figure OS-3 in Supplementary Material available online at https://doi.org/10.1155/2017/4614379) and without in others (Figure 3; Figures OS-4 and OS-5). The glucose stimulation and concomitant exposure to chemicals and glucose was always performed in KRBH buffer that did not contain phenol red (Figures 4 and 6).

### 2.3. Environmental Chemicals

To compare cellular effects induced by BPA and the phthalate metabolites MnBP, MiBP, and MEHP concentrations from 5 to 500 *μ*M were used since these affected several of the included endpoints in initial experiments. Stock solutions prepared in DMSO were used for the environmental chemical exposure. The maximum concentration of DMSO in the cell culture was 0.2%, and cells exposed to 0.2% DMSO only were used as negative controls. For the combinatory exposures, the 5 *μ*M concentration corresponds to exposure to 5 *μ*M of each of the chemicals and accordingly for the 50 and 500 *μ*M concentrations.

### 2.4. Cytotoxicity

Cellular viability was assessed by thiazolyl blue tetrazolium blue (MTT) assay as previously described [40]. In short, cells were incubated with 0.5 mg/mL MTT that is converted to dark blue, water-insoluble MTT formazan by mitochondrial dehydrogenases of living cells. This is subsequently solubilized by DMSO before measuring the absorbance at 570 nm, using a Galaxy Fluostar Optima plate reader (BMG Labtech, Offenburg, Germany). The viability was calculated by normalizing the absorbance to the control (DMSO only) in each experiment, by dividing the absorbance from the individual wells by the mean absorbance of all the control wells in that experiment.

To further investigate whether the environmental chemicals caused necrotic or apoptotic cell death the cells were stained for 30 min with the fluorescent DNA stains propidium iodide (PI; 10 *μ*g/mL) and Hoechst 33342 (5 *μ*g/mL). The fractions of viable, necrotic, and apoptotic cells were determined by fluorescence microscopy as Hoechst-positive cells with normal nuclei, PI-positive cells, and Hoechst-positive cells with condensed nuclei, respectively. Approximately 300 cells were counted for each sample.

### 2.5. Glucose-Induced Insulin Secretion

Insulin secretion from the INS-1E cells was assessed in Krebs Ringer bicarbonate HEPES (KRBH) buffer as described by Mergelen and coauthors (2004) [37]. Two different glucose-induced insulin secretion scenarios were applied: (i) exposure to environmental chemicals with a subsequent glucose exposure to assess chronic effects [24, 35, 41, 42] and (ii) simultaneous exposure to chemicals and glucose to investigate acute effects [35, 41, 43]. In the first exposure scenario, cells were exposed to environmental chemicals for 2, 24, 48, or 72 h, washed with 1 mL glucose-free KRBH, and incubated for 1 h with 1 mL glucose-free KRBH before a 30 min stimulation with 6.7 or 16.7 mM glucose in KRBH. For the simultaneous exposure scenario the cells were incubated with 1 mL glucose-free KRBH for 1 h followed by 2 h of concurrent exposure to environmental chemicals and either 6.7 or 16.7 mM glucose in KRBH. Since glucose concentrations between 5–7 mM and 15–17 mM are commonly used to measure glucose-induced insulin secretion [25, 37], a moderate (6.7 mM) and a high (16.7 mM) glucose concentration was included to reflect plasma glucose concentrations in healthy and diabetic/prediabetic individuals (T2D), respectively. At the end of the glucose exposure the supernatants were centrifuged for 10 min at 750 rpm to remove dead cells and stored at −70°C until analysis. The amount of released insulin was measured with a commercially available ELISA according to the manufacturer's manual. The color intensity was measured and quantified using a plate reader (TECAN Sunrise, Phoenix Research Products, CA, USA) with software (Magellan V 1.10).

In some experiments, 3-isobutyl-1-methylxanthine (IBMX; 25 *μ*M) and Forskolin (0.25 *μ*M) were included as positive controls for increased insulin secretion. The applied concentrations were chosen based on pilot studies including a range of concentrations reported in the literature [35, 44].

### 2.6. Cytokine-Induced Cell Death

Cytokine-induced *β*-cell death, one of the early events in the development of T1D, can be mimicked by exposure to a mixture of the proinflammatory cytokines interleukin- (IL-) 1*β*, interferon- (IFN-) *γ*, and tumor necrosis factor- (TNF-) *α* [14]. For this study, cytokine concentrations of 5 ng/mL IL-1*β*, 25 ng/mL IFN*γ*, and 25 ng/mL TNF*α* were chosen since these induced a medium effect on cell death in pilot studies. To investigate if the environmental chemicals altered the cellular sensitivity to cytokine-induced cell death, the cells were exposed to chemicals for 72 h, and the cytokine mixture was added to the cells the last 24 or 48 h of the chemical exposure.

### 2.7. Statistical Analysis

The statistical analysis was performed in GraphPad Prism (GraphPad Software, CA, USA). Two-way analysis of variance (ANOVA) was used to analyze the data sets, with Bonferroni post hoc tests to compare different treatment groups [45]. Data were obtained from 3-4 independent experiments, as indicated in the figure legends. Bars in columns are presented as mean ± standard error of mean (SEM) and *p* values < 0.05 are generally considered as significant.

## 3. Results

### 3.1. Viability and Toxicity

Long term treatment (72 h) with BPA alone or in combination with phthalate metabolites reduced the viability significantly at 50 *μ*M, while higher concentrations were necessary for phthalate metabolites alone (Figure 1(a)). Moreover, the viability was significantly lower after BPA exposure as compared to exposure to the corresponding concentration of MnBP, MiBP, and MEHP (indicated by # or *¤*  in Figure 1(a)). When additional concentrations were included in the 50 to 500 *μ*M range, the differential ability of BPA and MEHP to reduce cellular viability was even more evident (Figure 1(b)). The reduced viability was due to necrosis rather than apoptosis (data not shown), and BPA was significantly more potent with respect to induction of necrotic cell death at 24 h according to Hoechst/PI-analysis (Figure 1(c)), with similar percentages of necrotic cells after 72 h (data not shown).

As illustrated in Figure 1(a), the combined exposures reduced the viability to a similar extent as the most potent individual chemicals. When comparing the combined exposures and the calculated sum of the individual exposures the combinatory exposure appeared to induce additive effects for 50 *μ*M [46].

### 3.2. Insulin Secretion

Long term treatment (72 h) with environmental chemicals reduced the insulin secretion significantly, but only for the highest concentration of BPA and MEHP and the combinatory exposures (Figure 2). A correlation analysis showed a strong correlation between insulin secretion and viability (Online supplement 1, Figure OS-1). This decreased insulin release was therefore most likely due to cell death. Since BPA has been reported to increase the insulin release in a range of model systems, the insulin release from INS-1E cells was further assessed for different incubation times and glucose concentrations for BPA (5–100 *μ*M) and two positive controls, IBMX and Forskolin. For the low glucose concentration (6.7 mM), neither BPA nor the positive controls increased the insulin secretion after 2–48 h exposure, but BPA caused a nonsignificant reduction for 100 *μ*M BPA at 48 h (Figures 3(a)–3(c)). For the high glucose concentration (16.7 mM), 100 *μ*M BPA decreased the glucose-induced insulin secretion significantly for all exposure times, and the positive controls decreased the insulin secretion after 24 to 48 h of exposure, although not significantly for both compounds at all time points. For BPA this reduced insulin secretion was most likely linked to the reduced viability observed for 100 *μ*M (Figure 1, Figure OS-1). In contrast to BPA, the positive controls did not induce visible toxicity at the applied concentrations, as confirmed with the MTT assay in one experiment in triplicate (data not shown).

As positive controls, IBMX and Forskolin were expected to increase the insulin secretion rather than reduce it as observed (Figures 3(a)–3(c)). Since the cell growth medium contained 11.1 mM glucose, the 2–48 h incubation with positive controls in this medium could cause continuous insulin secretion. This could result in exhaustion of the cellular insulin secretion and ultimately reduced insulin secretion after the 30 min glucose stimulation (Figures 3(a)–3(c)). To test this, the insulin levels were measured in the cell culture medium after 48 h of stimulus incubation, by harvesting the medium prior to the 30 min of glucose challenge. Indeed, the insulin secretion was significantly increased after IBMX and Forskolin exposure (Figure 3(d)). Thus, the positive controls appeared to exhaust the cellular insulin secretion in response to the prolonged exposure to 11 mM glucose in the cell culture medium. In contrast, 100 *μ*M BPA reduced insulin levels in the medium significantly after 48 h exposure, consistent with the toxicity observed after 72 h exposure for this concentration.

Since the positive controls failed to increase the insulin secretion in the first exposure scenario, a concomitant exposure scenario was also tested, where INS-1E cells were exposed to chemicals and glucose concomitantly for 2 h. In this exposure scenario, both positive controls increased the insulin secretion at both glucose concentrations (Figure 4). In contrast, BPA only increased the insulin secretion at the low glucose concentration, for 50 and 100 *μ*M BPA (Figure 4(a)).

The influence on the glucose-induced insulin secretion was tested for the phthalate metabolites MnBP, MiBP, and MEHP in concentrations between 0.5 and 100 *μ*M in the exposure scenario that was most sensitive to BPA-induced effects, a 2 h concomitant exposure at the low glucose concentration. At 100 *μ*M, MnBP and MEHP increased the insulin secretion significantly, while MiBP had a weak nonsignificant effect (Figure 4(b)). MEHP induced a significantly higher increase in insulin secretion than MnBP. The response induced by MEHP was slightly higher than the response induced by the positive control IBMX. In comparison, the response induced by 100 *μ*M BPA was approximately twice as high as the IBMX response (Figure 4(a)).

### 3.3. Sensitivity to Cytokine-Induced Cell Death

To examine if environmental chemicals increased the *β*-cell susceptibility to cytokine-induced cell death, cell viability was measured by MTT after exposure to INF-*γ*, TNF-*α*, and IL-1*β* the last 48 h of the chemical exposure. The 48 h cytokine treatment caused approximately 50% necrosis (Online supplement 1, Figure OS-2), and after either 24 or 48 h cytokine treatment very few apoptotic cells were observed. The percentages of necrotic cells were generally in agreement with the observed reductions in viability measured by MTT assay. The environmental chemicals at concentrations of 50 and 500 *μ*M reduced the viability in the presence of cytokines (Figure 5(a)), with a similar response pattern and magnitude as in absence of cytokines (Figure 1(a)). This is visualized in Figure 5(b), where the data from Figures 5(a) and 1(a) are plotted in the same graph, showing effects in the absence (blank bars) or presence (stripes) of cytokines relative to their respective controls. Notably, as expected, the cytokine treatment itself caused a time dependent reduction in the viability, with approximately 45% viable cells after 48 h as seen in Online supplement 1 (Figure OS-2).

When comparing the effects of combinatory exposure to the calculated sum of the effects induced by the individual exposures, an additive trend was observed, as for the effects of environmental chemicals alone [46].

### 3.4. Environmentally Relevant Chemical Concentrations

BPA concentrations with relevance to human exposure levels (10–500 nM) did not affect the insulin release during concomitant exposure with either medium or high glucose concentrations (Figure 6). Similarly, 72 h of incubation with environmentally relevant BPA and phthalate metabolite concentrations (1–500 nM) did not affect viability, insulin secretion, or cytokine-induced cell death significantly (Online supplement 1, Figure OS-3). However, a trend towards a reduced insulin secretion was observed for the lowest chemical concentrations (1–50 nM; U-shaped response), resulting in a significant overall effect of chemical concentration in the ANOVA analysis (Figure OS-3). The insulin secretion and viability were not correlated with these low chemical concentrations (data not shown).

## 4. Discussion

The use of BPA and phthalates in a large variety of consumer products has resulted in widespread human exposure. Although epidemiological studies have suggested a link between exposure to these environmental chemicals and diabetes [12, 17–19, 30–32], the question of causality remains controversial. Several studies suggest an impact of BPA on *β*-cell function with relevance to T2D [20, 24–26] or accelerated development of T1D in NOD mice [23, 28], while less is known about effects of phthalates on diabetes-related endpoints [22, 33–35]. In this study, BPA was more potent than the three phthalate metabolites MnBP, MiBP, and MEHP in inducing reduced viability and increased insulin release from the pancreatic *β*-cells INS-1E, while susceptibility to cytokine-induced cell death was not affected.

The serum concentrations of BPA and MEHP reported in human biomonitoring studies are in the ranges 1–120 nM and 1–1700 nM, respectively (summarized in [46]). When INS-1E cells were exposed to such low concentrations of BPA and phthalate metabolites (1–500 nM) for 72 h, their viability was not affected. In contrast, concentrations in the micromolar range were necessary to reduced cellular viability, 50 and 100 *μ*M for BPA and MEHP, respectively. This was somewhat surprising since BPA concentrations in the nM range have been reported to induce a range of toxic effects in *β*-cells including apoptosis and mitochondrial swelling and dysfunction [24, 43, 47]. Moreover, a recent study by Lin and coauthors in a similar cell line (INS-1) showed decreased cell viability after BPA exposure as low as 200 nM for 12 h, with an increased rate of apoptosis in a dose dependent manner [42]. However, using the same cell line as Lin and coauthors (INS-1), we could not reproduce similar, low-concentration effects of BPA in our lab, as much higher BPA concentrations (50 *μ*M) were necessary to reduce the INS-1 cell viability significantly (Online supplement 1, Figure OS-4), and even for this concentration BPA only reduced the viability with approximately 20%. Thus, in our lab both the INS-1 and INS-1E cell lines appear to have a very similar, relatively low sensitivity to BPA.

T2D is characterized by a relative or absolute deficiency in insulin secretion, but during disease development hypersecretion of insulin may occur as an early step in the process leading to *β*-cell failure [16]. BPA has been reported to increase glucose-induced insulin secretion from pancreatic islets, with mitochondrial dysfunction as a suggested mechanism [24, 25, 42]. In the INS-1E cells, the glucose-induced insulin secretion was significantly reduced after 72 h exposure to 500 *μ*M of BPA, MEHP, and the combinatory exposures. This was most likely due to the chemical-induced toxicity (reduced viability) observed at these concentrations. However, 2 h of concomitant exposure to 50 and 100 *μ*M BPA and glucose increased insulin secretion significantly, in accordance with the literature on BPA-induced insulin hypersecretion. The increased insulin release was only observed for the low glucose concentration (6.7 mM), in accordance with the data from Hectors and coauthors (2013) [35]. In contrast, BPA-induced effects on insulin secretion or cellular insulin content were more evident during high than low glucose concentrations (larger magnitude of effect or effects at lower concentrations) in some model systems [24, 25, 42], whereas others report similar effects for different glucose concentrations [43]. Thus, the impact of glucose concentration in the BPA-induced effects on insulin secretion seems to depend on the specific model system applied.

When BPA concentrations with relevance to human exposures were tested (10–500 nM), concomitant exposure with glucose did not affect the insulin release. This was in contrast to previous studies showing that BPA in the nM range was able to increase insulin secretion in both *β*-cell lines and primary cells [24, 35, 41–43, 48]. Other subclones of the INS-1 cell line (INS-1 and INS-1 832/13) have also been reported to be sensitive to lower concentrations of BPA in terms of glucose-induced insulin secretion than our INS-1E cells [35, 42]. However, a similar (low) sensitivity to BPA was seen for both INS-1 and INS-1E cells, also for insulin secretion, in our laboratory (Online supplement 1, Figure OS-5). A role for estrogen receptors *α* and *β* in the BPA-induced effects observed in pancreatic islets has been suggested in the literature [48, 49]. Interestingly, the INS-1 cells appear to have a low sensitivity to estradiol-induced effects, even during overexpression of estrogen receptor [50]. Thus, it is tempting to speculate that the low sensitivity to environmental chemicals of some of the INS-1 clones may be due to their low sensitivity to estrogen-induced effects.

During preexposure to chemicals for 2 to 48 h, followed by separate glucose stimulation, the glucose-induced insulin release was generally decreased rather than increased (Figure 3). The unexpected reduction in insulin secretion in response to the positive controls IBMX and Forskolin could be explained by exhaustion of the cellular insulin levels during the 2–48 h incubation with cell culture medium containing 11.1 mM glucose. In contrast, the reduced insulin levels in the medium after 48 h of BPA exposure, suggested a different explanation for the BPA-induced reduction in the glucose-induced insulin release at this exposure time. Although cellular viability was not measured after these exposures, it is tempting to speculate that the reduced insulin release after the glucose challenge was caused by reduced viability, as for the 72 h BPA exposure.

The possible link between phthalate exposure and T2D has received increasing attention in epidemiological studies [12, 17, 30, 31], but there are still relatively few experimental studies addressing the effects of phthalates on endpoints with relevance to diabetes development [22, 33–36]. When the phthalate metabolites were tested in the exposure scenario where BPA increased the glucose-induced insulin release, all three phthalate metabolites had a lower impact on the insulin release than BPA, and the potency of the phthalate metabolites were MEHP > MnBP > MiBP. Hectors and coauthors reported that much higher concentrations of DEHP were required to increase insulin release from an INS-1 clone in comparison to BPA (100 *μ*M versus 10–100 nM) [35]. Although, our data suggest that phthalate metabolites are less potent than BPA in affecting *β*-cell function, the concentrations of some phthalate metabolites in serum may be considerably higher than the BPA concentrations [46]. In particular, the maximum reported levels of MEHP and MiBP were 15 and 80 times higher than BPA, respectively. Thus, further studies of the impact of phthalates on *β*-cell function are warranted in more sensitive cell types and model systems. Interestingly, another phthalate metabolite MEP was recently shown to increase insulin release from 1.1B4 human pancreatic *β*-cells after 24 h exposure to nM concentrations, with PPAR*γ* and ER*α* as suggested mechanisms [36]. This cell line may emerge as a novel sensitive model system for mechanistic studies of how environmental chemicals affect insulin secretion [36, 51].

Since cytokine-induced *β*-cell death seems to be involved in the development of T1D, we also examined whether environmental chemicals could increase the *β*-cell sensitivity to cytokine-induced cell death* in vitro*. Overall, the environmental chemicals did not increase the *β*-cells sensitivity to cytokine-induced cell death. The observed reduction in viability after cytokine exposure alone, of approximately 30 and 65% after 24 and 48 h, respectively, suggested a strong cytokine-induced cytotoxicity. Hoechst/PI analyses showed that this reduced viability was primarily dominated by necrosis with <1% apoptosis, similar to the percentage observed for the high concentrations of the environmental chemicals. There appears to be a general agreement that cytokines have a direct role in inducing pancreatic *β*-cell death leading to a decrease in *β*-cell viability in diabetes development, but whether this cell death occurs by necrosis, apoptosis, or both is still debated [52–54]. Our data, suggesting that cytokine-induced *β*-cell death is dominated by necrosis, differ from some prior studies that claim an important contribution from apoptosis [55, 56]. On the other hand, some studies in INS-1 cells and isolated islets support that necrosis is the predominant type of cell death by cytokine-induced killing of *β*-cells [53, 57, 58]. A factor that might contribute to these conflicting results with respect to type of cell death could be differences in the applied cytokine concentrations, and it has been postulated that the dose of cytokines might determine which cell death pathway is preferentially activated [59].

Phenol red is a standard ingredient in most cell culture media that has been suggested to induce estrogenic effects [60]. Presently, the environmental chemical exposure was performed in presence of phenol red (72 h exposure; Figures 1, 2, and 5 and OS-3) or in its absence (2–48 h exposure in Figure 3 or exposure in KRBH buffer in Figures 4 and 6). However, the presence or absence of phenol red did not seem to have a major impact on the insulin secretion (Figure 2 versus Figure 3; although different chemical exposure times were tested). Moreover, the potential impact of phenol red on cell line models is highly controversial [61–63] and a comparative study in nine estrogen receptor-positive cell lines concluded that phenol red in culture medium was insufficient to cause estrogenic effects [64]. Thus, the presence of phenol red in some of the current experiments does not seem to explain the lack of effect of low chemical concentrations in the INS-1E cells.

Most studies of BPA-induced effects on *β*-cells in the literature present data either for insulin secretion [24, 25, 35, 43, 65] or cellular insulin content [42, 48]. The one study assessing both endpoints, only reported effects of BPA on insulin content. In our study, cellular insulin content was only measured in an initial experiment after 48 h exposure to 1–100 nM BPA, where no profound effects were observed. Since the level of secreted insulin is what ultimately has consequences for the surrounding cells and tissues, this was chosen as the study endpoint rather than insulin content. However, a possible greater impact of environmental chemicals on the cellular insulin content than its secretion in the present model system cannot be completely excluded.

## 5. Conclusion

BPA was more potent than the investigated phthalate metabolites with respect to affecting insulin secretion and viability in INS-1E cells. However, as phthalate metabolites may be present in considerably higher concentrations than BPA in human serum, further studies of the impact of phthalates on *β*-cell function are warranted in more sensitive cell types and model systems.

## Supplementary Material

The online supplement contains supplementary data for (1) correlation of insulin secretion and cellular viability, (2) cytokine-induced cytotoxicity, (3) 72h exposure to environmentally relevant concentrations of BPA and phthalate, and (4) effects of BPA on viability and insulin secretion in INS-1 cells.
