# Supplementary material for: Bisphenol A Is More Potent than Phthalate Metabolites in Reducing Pancreatic β-Cell Function
Source: Biomed Res Int. 2017 Feb 13;2017:4614379. doi: 10.1155/2017/4614379 (PMC5327753; doi:10.1155/2017/4614379)
Supplement: Supplementary file 1 — The online supplement contains supplementary data for (1) correlation of insulin secretion and cellular viability, (2) cytokine-induced cytotoxicity, (3) 72h exposure to environmentally relevant concentrations of BPA and phthalate, and (4) effects of BPA on viability and insulin secretion in INS-1 cells. [file 4614379.f1.docx]

**Online supplement 1:**

This online supplement contains supplementary data for (1) correlation of insulin secretion and cellular viability, (2) cytokine-induced cytotoxicity, (3) lack of effects of 72h exposure to environmentally relevant BPA and phthalate concentrations and (4) effects of BPA on viability and insulin secretion in INS-1 cells.

1. **Correlation of insulin secretion and cellular viability of INS-1E cells.**

As illustrated in Figure OS-1, there was a strong correlation between group mean insulin secretion (Figure 2 in the main manuscript) and viability (Figure 1a), when including all chemical exposures and controls. This supports that the reduced insulin secretion after chemical exposure was due to reduced viability.


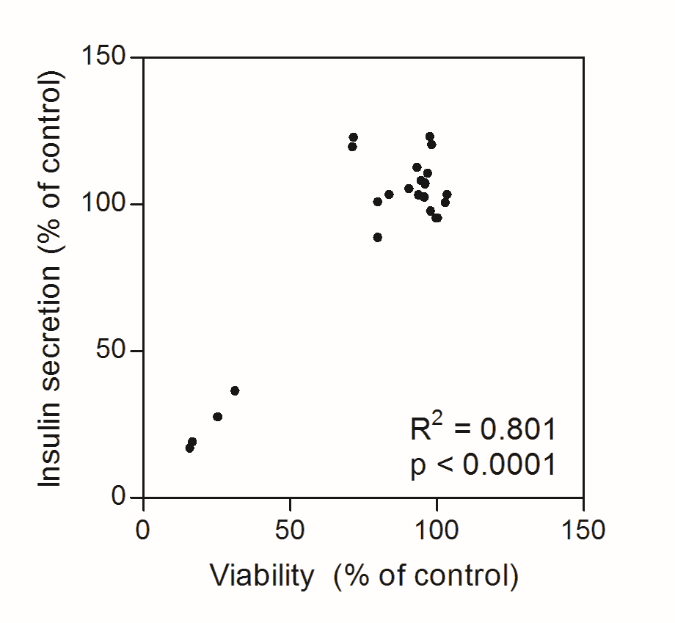


**Figure OS-1: Correlation of insulin secretion and viability.** Each dot in the plot represents the mean response, i.e. viability or insulin secretion to a single chemical concentration (corresponding to bar height in Figure 1a and 2 in the main manuscript). The data were analyzed by Pearson correlation in GraphPad Prism.

1. **Effects of the pro-inflammatory cytokines IL-1β, TNFα and INFγ on viability and toxicity in INS-1E cells.**

As illustrated in Figure OS-2, 24 or 48h incubation with the pro-inflammatory cytokines IL-1β, TNFα and INFγ reduced the viability of INS-1E cells, as measured with the MTT assay. The effect of cytokine incubation increased with exposure time. Accordingly, the percentage of necrotic and apoptotic cells only increased significantly for the 48h cytokine exposure. The percentage of apoptotic cells was very low, generally below 2%, thus the cytokine induced cell death was dominated by necrosis.

**
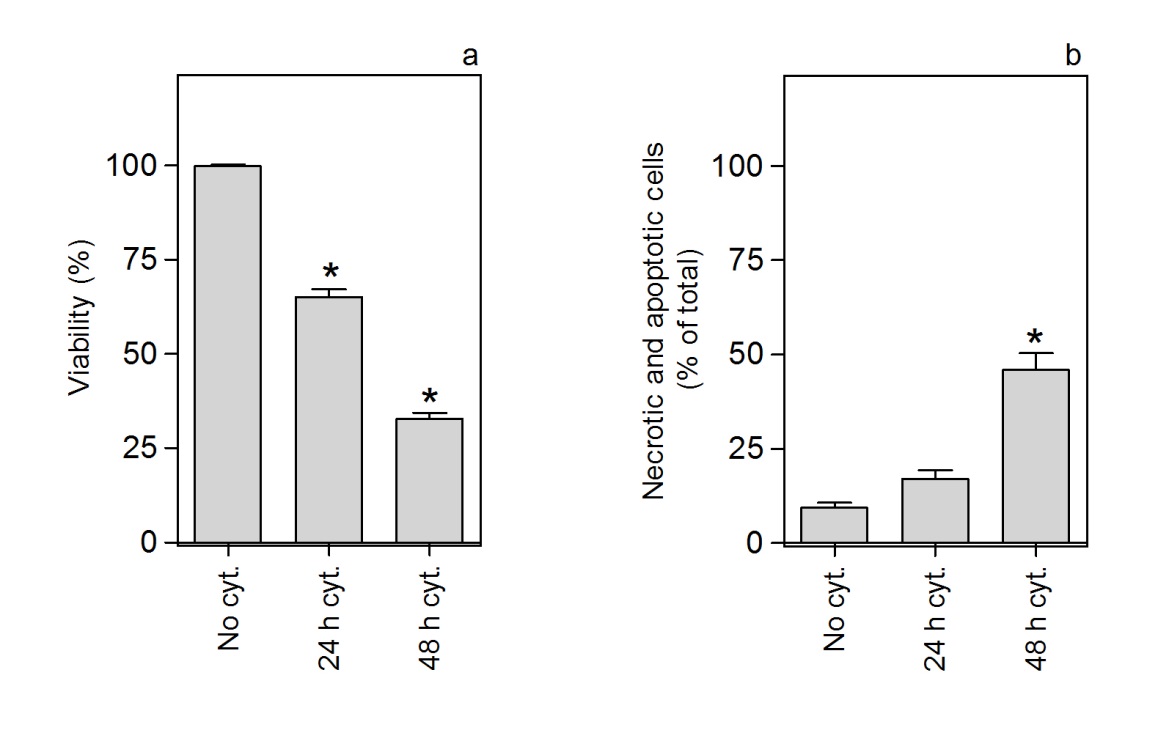
**

**Figure OS-2: Effect of pro-inflammatory cytokines on viability and necrosis.** INS-1E cells were incubated with the pro-inflammatory cytokines IL-1β (5 ng/ml), TNFα (25 ng/ml) and INFγ (25 ng/ml) the last 24 or 48 h of exposure. In a) the cell viability was measured by the MTT assay and normalized to controls not exposed to cytokines, while b) shows the percentage of necrotic cells after fluorescence microscopy of Hoechst/PI stained cells. * indicates significant difference from respective controls (two-way ANOVA with Bonferroni post-test, N=3).

1. **72 h exposure to environmentally relevant chemical concentrations in INS-1 E cells.**

When 1-500 nM BPA or phthalate metabolites were tested alone or in combination, a 72h exposure did not affect viability, insulin release or cytokine-induced cytotoxicity significantly (Figure OS-3). Although no significant differences were detected in the post-hoc tests of pairwise comparisons between the concentration groups within each chemical, a trend towards a reduced insulin secretion was observed for all the low chemical concentrations (U-shaped response), resulting in a significant overall effect of the chemical concentration in the ANOVA analysis (Figure OS-3). The reduced insulin secretion did not correlate with the reduced viability (data not shown).

**
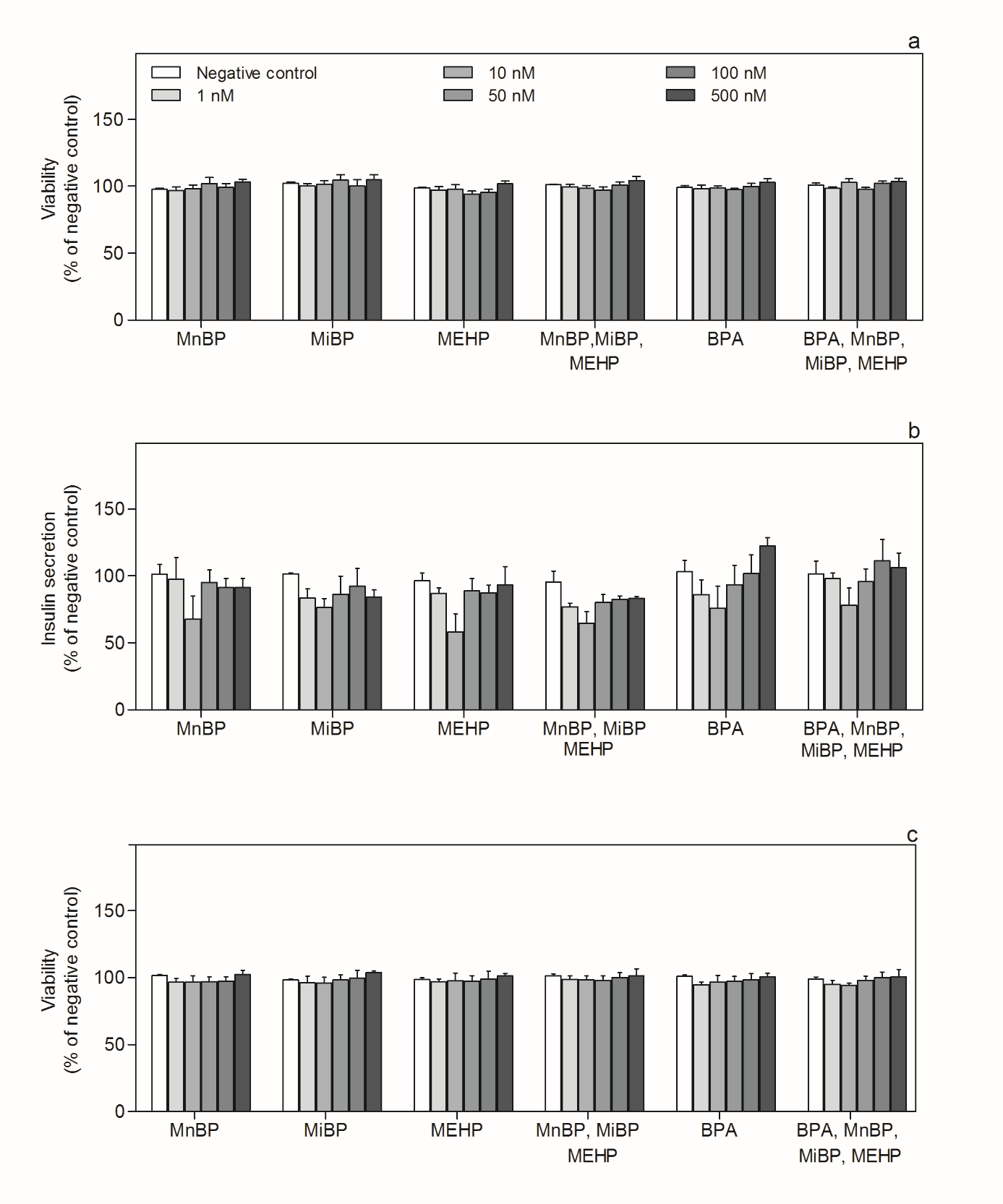
**

**Figure OS-3: No effect of 72 h exposure to environmentally relevant chemical concentrations.** INS-1E cells were incubated with the indicated concentrations of environmental chemicals for 72 h to measure a) cell viability by the MTT assay, b) glucose-induced insulin secretion (1h incubation with glucose free KRBH buffer followed by 30 min stimulation with 6.7 mM glucose in KRBH buffer) and c) cytokine-induced cell death (exposure to the pro-inflammatory cytokines IL-1β (5 ng/ml), TNFα (25 ng/ml) and INFγ (25 ng/ml) the last 48 h of exposure). In the combinatory exposures the indicated doses reflect concentrations of each chemical rather than the total (additive) chemical concentration. The graph shows normalized data, e.g. divided by the mean value of the negative controls in that experiment. The data were analyzed by 2-way ANOVA with the two factors ‘chemical’ and ‘concentration’, and with Bonferroni post-hoc test to compare each chemical concentration to its respective control (N=3-4). In a-b), the ANOVA analysis showed a significant overall effect of the ‘concentration’ factor, but no significant differences between groups in the post-hoc tests.

1. **INS-1 cells showed similar responsiveness to BPA as INS-1E cells**

Since exposure to 10-500 nM BPA was recently reported to induce toxicity and increased insulin release in INS-1 cells [1], the sensitivity of these cells were compared to that of the INS-1E cells in our lab. The INS-1 cells (Addexbio Technologies, CA, USA), were cultured according to the manufacturers specifications; in a humidified atmosphere at 37°C and 5% CO_2_ in RPMI 1640 medium supplemented with 5% heat-inactivated fetal calf serum, 1 mM sodium pyruvate, 50 μM 2-mercaptoethanol, 2 mM glutamine, 10 mM HEPES, 100 U/ml penicillin, and 100 μg/ml streptomycin. The concomitant incubations with BPA and glucose were performed as described for the INS-1E cell line in the main manuscript. In contrast to the data reported by Lin and co-authors [1], the low BPA concentrations in the nM range did not affect viability (Figure OS-4) or insulin release (Figure OS-5). On the contrary, the responses induced in the INS-1 cells showed a similar response pattern as that observed in the INS-1E cells, with reduced viability and increased insulin release after exposure to 50 µM BPA (Figure 1 and 4 in the main manuscript, respectively).


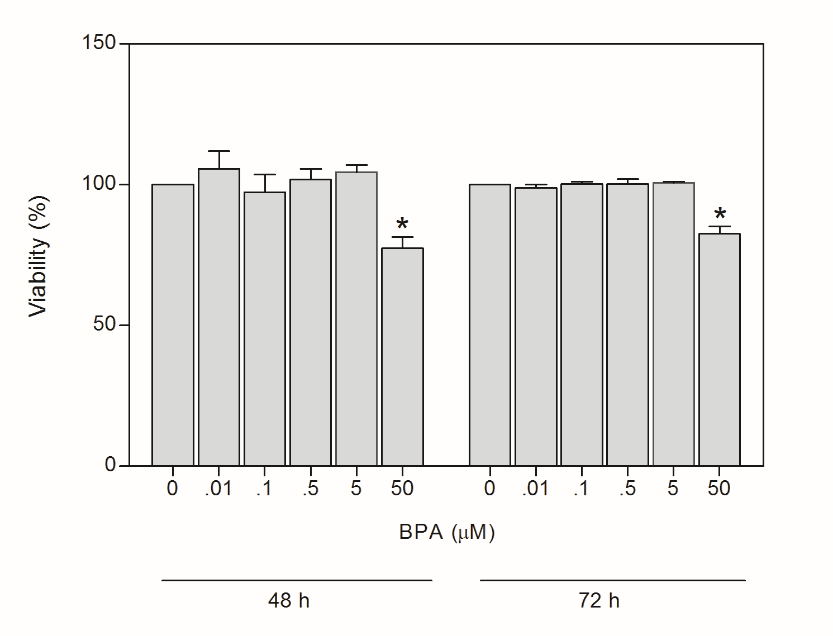


**Figure OS-4:** INS-1 cells were incubated with the indicated concentrations of BPA for 48 and 72h. The cell viability was measured by MTT and normalized to control*.* * indicates significant decreases compared to control (One sample t-test, N=3-4).


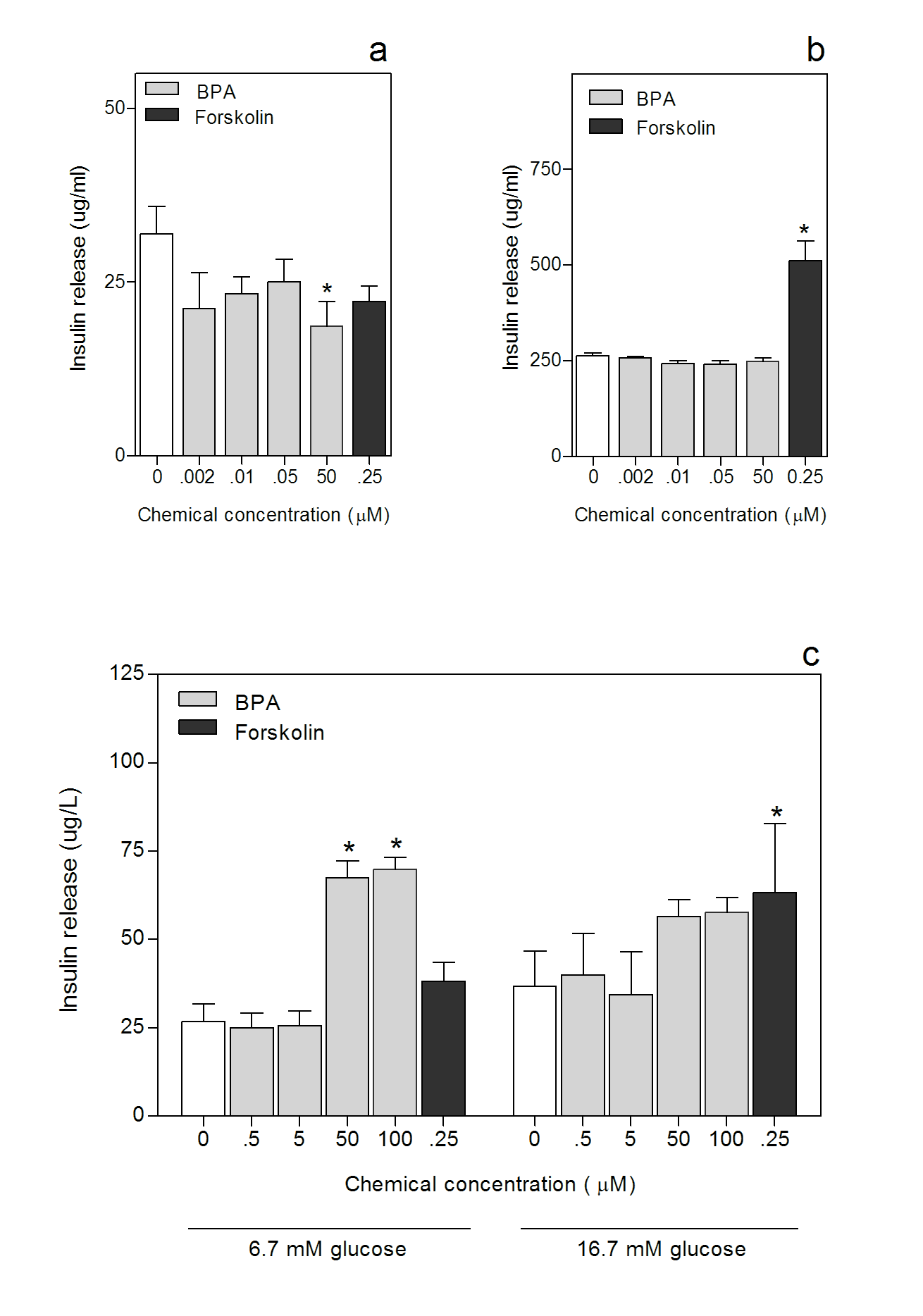


**Figure OS-5:** INS-1 cells were incubated with the indicated concentrations of BPA or the positive control Forskolin for 48h before a 1h incubation with glucose free KRBH buffer and a 30 min incubation with 16.7 mM glucose in KRBH buffer. a) shows the insulin levels in the KRBH buffer, while b) shows the levels of insulin in the medium after the 48h exposure. * indicates significantly different from control (1-way ANOVA with Dunnets post test, N=4). c) INS-1 cells were incubated with the indicated concentrations of BPA or the positive control Forskolin and 6.7 or 16.7 mM glucose in KRBH buffer for 2h, after a 1h incubation with glucose free KRBH buffer. * indicates significant increase compared to control (2-way ANOVA, repeated measures, with Bonferroni post test, N=3).

**References**

1. Lin Y, Sun X, Qiu L, et al. Exposure to bisphenol A induces dysfunction of insulin secretion and apoptosis through the damage of mitochondria in rat insulinoma (INS-1) cells. Cell Death Dis. 2013;17(4):206.
